# Increased dental visits in patients with rheumatoid arthritis: a secondary cohort analysis of population based claims data

**DOI:** 10.1186/s12903-022-02661-w

**Published:** 2022-12-15

**Authors:** Ching-Ya Juan, Chia-Wen Hsu, Ming-Chi Lu

**Affiliations:** 1 Division of Dentistry, Dalin Tzu Chi Hospital, Buddhist Tzu Chi Medical Foundation, Dalin, Chiayi Taiwan; 2 Department of Medical Research, Dalin Tzu Chi Hospital, Buddhist Tzu Chi Medical Foundation, Dalin, Chiayi Taiwan; 3 Division of Allergy, Immunology and Rheumatology, Dalin Tzu Chi Hospital, Buddhist Tzu Chi Medical Foundation, No. 2, Minsheng Road, Dalin, Chiayi 62247 Taiwan; 4 School of Medicine, Tzu Chi University, Hualien, Taiwan

**Keywords:** Rheumatoid arthritis, Dental disorders, Dental caries, Pulpitis, Gingivitis, Periodontitis, Oral ulcer

## Abstract

**Objective:**

To study the utilization of dental care in patients with rheumatoid arthritis (RA) and compare the incidence of common dental disorders in patients with and without RA.

**Methods:**

This data used in this study was from the population-based Taiwan's National Health Insurance Research Database. We identified 1337 patients with newly diagnosed RA between January 2000 and December 2012. We also identified 13,370 individual without a diagnosis of RA using frequency matching on 5-year age intervals, sex, and index year. Patients with a diagnosis of primary Sjögren's syndrome were excluded. Dental disorders were identified using respective ICD-9-CM codes confirmed by dentists. The incidence and incidence rate ratio [IRR] of each dental disorders were calculated using Poisson regression.

**Results:**

Compared with the comparison cohort, the prevalence of dentist visits in the RA cohort were significantly higher (70.3% vs. 66.7%, *p* = 0.008) and the frequency of dentist visits in the RA cohort were also significantly higher (median 2.67 vs. 1.78 per year, *p* < 0.001). In addition, the incidence of visits for dental caries (adjusted IRR 1.16, *p* < 0.001), pulpitis (adjusted IRR 1.12, *p* = 0.044), gingivitis (adjusted IRR 1.13, *p* = 0.027), periodontitis (adjusted IRR 1.13, *p* = 0.004), and oral ulcer (adjusted IRR 1.24, *p* = 0.003) were higher in patients with RA.

**Conclusions:**

An elevated prevalence and frequency of dental visits were associated with patients with RA. In addition, elevated incidence of dental disorders, including dental caries, pulpitis, gingivitis, periodontitis, and oral ulceration, were observed. Oral health should be accessed regularly in patients with RA.

## Introduction

Systemic diseases can affect oral health and studies have shown that many systemic diseases, including rheumatoid arthritis (RA) are closely associated with dental disorder [1]. RA is a common systemic autoimmune disease characterized by persistent joint inflammation leading to increased mortality. The prevalence of RA was estimated to be between 0.5 and 1.0% with a female to male ratio of approximately 2.5:1 [2]. In Taiwan, the prevalence of RA was reported to be 0.26–0.93% [3]. RA can affect not only the joints, but it can also involve other organ systems, such as the lung, skin vasculitis, and lead to systemic comorbidities [4, 5]. Among these comorbidities or extra-articular manifestations, dental disorders are common in patients with RA, which would increase the need of treatment from dental professionals [6].

Among dental disorders, it is well known that patients with periodontitis have an increased risk of developing RA, and that patients with RA also showed an increased risk of developing periodontitis [7, 8]. Martinez-Martinez et al. reported that the frequency and severity of dental caries were higher in patients with RA compared with controls [9]. In addition, the prevalence of temporomandibular disorders was found to be higher in patients with RA [10, 11]. However, the prevalence and incidence of common dental disorders, such as dental caries, pulpitis, oral ulcer, and stomatitis in patients with RA, was not clear. We hypothesized that the incidence of common dental disorders is higher in patients with RA. In this study, we compared the prevalence of dental conditions and the frequency of dental visits in patients with or without RA. We also performed separate analyses to assess the incidence and incidence rate ratios (IRRs) of common dental disorders.

## Materials and methods

### Identification of the RA cohort and a comparison cohort

This is a secondary cohort analysis based on the claim data from Taiwan's National Health Insurance Research Database (NHIRD). The study design was based on our previous study with modifications [12]. The study protocol was reviewed and approved by the institutional review board of Dalin Tzu Chi Hospital, Buddhist Tzu Chi Medical Foundation, Taiwan (No.B10004021). The need for informed consent from individuals was waived because the data in NHIRD were deidentified.

Patients with newly diagnosed RA were identified based on the International Classification of Diseases, Ninth revision, clinical modification (ICD-9-CM) code 714.0 (rheumatoid arthritis) using the 2000–2012 catastrophic illness datafile of the NHIRD. RA is considered a catastrophic illness in Taiwan. Patients with RA can apply for a certificate from the National Health Insurance Administration (NHIA), and their medical records, serological, pathological, and imaging reports will be reviewed by at least two specialists. If approved, holders of a RA catastrophic illness certificate are exempted from copayments for health care expenses related to the treatment of RA. The diagnosed of RA was based on the American Rheumatism Association 1987 revised criteria for the classification of rheumatoid arthritis [13]. The date of application for the catastrophic illness certificate was defined as the index date for RA in this study. Patients whose ages less than 20 years, more than 80 years on the index date or with a diagnosis of primary Sjögren’s syndrome, defined by the catastrophic illness datafile, were excluded from the study. The ending year of the dataset was 2012 because of a change in data protection policy in Taiwan.

A comparison cohort was constructed based on a random sample of the outpatient datafile of the 2000 Longitudinal Health Insurance Database (LHID 2000) of the NHIRD. In this study, 10 patients were selected, based on frequency matching for sex, 5-year age interval, and index year, for each patient with RA to form a comparison cohort. In the comparison cohort, those ages less than 20 years, more than 80 years on the index date, or with a diagnosis of primary Sjögren’s syndrome, defined by the catastrophic illness datafile, were excluded from the study.

A number of diseases were identified as potential confounders, including hypertension, diabetes mellitus, congestive heart failure, chronic pulmonary disease, malignancy, dyslipidemia, coronary artery disease, myocardial infraction, peripheral vascular disease, cerebrovascular disease, dementia, musculoskeletal disorders, peptic ulcer disease, chronic kidney disease***,*** and liver disease. These diseases were identified by their respective ICD-9-CM codes and inclusion criteria as in our previous study [12].

### Identification of dental disorders

The RA cohort and the comparison cohort were followed until our outcome of interest had occurred or when the end of the follow-up period had reached. Only dental visits occurred at least 90 days after the index date were included in the analysis. Various dental disorders of patients with RA were reviewed according to ICD-9-CM codes. We selected common or potentially RA-related dental disorders based on the frequency of dental visits and they included: (1) periodontitis (ICD-9 codes: 523.3, 523.4, 523.5, and 523.8) (2) dental caries (ICD-9 code: 521.0), (3) gingivitis (ICD-9 codes: 523.0, 523.1, and 523.2), (4) pulpitis (ICD-9 code: 522.0), (5) oral ulceration (ICD-9 code: 528.2)**,** (6) stomatitis (ICD-9 code: 528.0), and (7) temporomandibular joint disorders (ICD-9 code: 524.6). The incidence and IRRs for each of these dental disorders were separately calculated in the RA cohort and the comparison cohort. The follow-up duration used for the calculation of person-years was obtained by subtracting the date when the outcome of interest occurred from the index date. Patients who were diagnosed with the outcome of interest within 90 days before the index date were excluded from the calculation of the incidence and IRRs.

### Statistical analysis

Demographic data, the prevalence and frequency of the visits due to specific dental disorders between the RA cohort and the comparison cohort were compared using the Chi-square test, the *t*-test, or the non-parametric Mann–Whitney *U*-test, as appropriate. The incidence per 1000 person-years was calculated separately for the RA cohort and the comparison cohort. Poisson regression models using person-years as the offset variable, with or without adjusting for potential confounding variables were used to calculate IRRs for the outcome variables. All analyses were conducted with IBM SPSS Statistics for Windows, version 24.0 (IBM Corp, Armonk, NY, USA). A p-value less than 0.05 was considered statistically significant.

## Results

### Basic characteristics and comorbidities of patients in the RA cohort and in the comparison cohort

We selected 1337 newly-diagnosed patients with RA and 13,370 patients without RA in this study. There were no significant differences in sex, age, and geographic region between the two cohorts. In Table 1, the insurance premium levels were significantly higher in the RA cohort. Patients with RA showed a significantly higher proportion of chronic pulmonary diseases (3.0% vs. 2.0%; *p* = 0.019), musculoskeletal disorders (4.5% vs. 0.4%; *p* < 0.001), and peptic ulcer diseases (5.1% vs. 2.5%; *p* < 0.001), but a significantly lower proportion of cancer (1.0% vs. 1.8%; *p* = 0.033) compared with those in the comparison cohort.

**Table 1:** Basic characteristics of the rheumatoid arthritis cohort and the comparison cohort (N = 14,707)

Variable	N (%)				*p* value
	Rheumatoid arthritis cohort 1337 (9.1)		Comparison cohort 13,370 (90.9)		
Sex					> 0.999
Male	302	(22.6)	3020	(22.6)	
Female	1035	(77.4)	10,350	(77.4)	
Age interval at entry (years)					> 0.999
20–54	721	(53.9)	7210	(53.9)	
> 55	616	(46.1)	6160	(46.1)	
Mean age (standard deviation), years	53.2	(13.4)	53.2	(13.4)	> 0.999
Median (lower and upper quartile), years	53.0	(44.0, 63.0)	53.0	(44.0, 63.0)	
Salary grades for insurance (n = 14,689)					< 0.001
≤ 19,000	611	(46.0)	7203	(53.9)	
19,001–24,000	483	(36.4)	4115	(30.8)	
≥ 24,001	234	(17.6)	2043	(15.3)	
Geographic region (n = 14,241)					0.254
Northern	748	(58.1)	7880	(60.8)	
Central	234	(18.2)	2140	(16.5)	
Southern	278	(21.6)	2653	(20.5)	
Eastern	28	(2.2)	280	(2.2)	
Comorbidities					
Hypertension	106	(7.9)	1022	(7.6)	0.710
Diabetes	52	(3.9)	580	(4.3)	0.440
Congestive heart failure	8	(0.6)	62	(0.5)	0.495
Chronic pulmonary disease	40	(3.0)	271	(2.0)	0.019
Cancer	13	(1.0)	235	(1.8)	0.033
Dyslipidemia	40	(3.0)	327	(2.4)	0.222
Coronary artery disease	3	(0.2)	46	(0.3)	0.469
Prior myocardial infarction	1	(0.1)	10	(0.1)	> 0.999
Peripheral vascular disease	1	(0.1)	18	(0.1)	0.561
Cerebrovascular disease	25	(1.9)	230	(1.7)	0.689
Dementia	1	(0.1)	35	(0.3)	0.187
Musculoskeletal disorders^a^	60	(4.5)	50	(0.4)	< 0.001
Peptic ulcer disease	68	(5.1)	337	(2.5)	< 0.001
Chronic kidney disease	16	(1.2)	114	(0.9)	0.200
Liver disease	22	(1.6)	174	(1.3)	0.296

Socioeconomic status was estimated by insurance premiums based on salary. Low: < 19,000 New Taiwan dollars (NT$); middle: 19,001–24,000; and high: > 24,000

*P* values were obtained by Chi-square test for categorical variables and *t*-test or Mann–Whitney *U*-test for continuous variables, as appropriate

^a^Excluding the diagnosis of rheumatoid arthritis (ICD-9 code: 714.0)

### Prevalence, frequency, and common causes of dental visits

Table 2 showed that patients with RA had a higher prevalence (70.3% vs. 66.7%, *p* = 0.008) and frequency (medium 2.67 vs. 1.78 per year, *p* < 0.001) of dental visits compared with those without RA. The prevalence and frequency of dental visits for dental caries, pulpitis, gingivitis, periodontitis, and oral ulceration, but not stomatitis were found to be significantly higher in the RA cohort. Moreover, the prevalence and frequency of dental visits for temporomandibular joint disorders were slightly higher in the RA cohort with marginal significance (1.6% vs. 1.0%, *p* = 0.065).

**Table 2:** The prevalence and frequency of dental disorders in the rheumatoid arthritis and comparison cohort (N = 14,707)

Variable	N (%)				*p* value
	Rheumatoid arthritis cohort 1337 (9.1)		Comparison cohort 13,370 (90.9)		
Dental visits					
Prevalence (%)	940	(70.3)	8921	(66.7)	0.008
Number of visits, median (Q1, Q3) (per year)	2.67	(0.0, 14.2)	1.78	(0.0, 10.9)	< 0.001
Number of visits, mean (SD) (per year)	14.25	(28.87)	11.79	(26.56)	
Dental caries (ICD-9-CM 521.0)					
Prevalence (%)	747	(55.9)	6885	(51.5)	0.002
Number of visits, median (Q1, Q3) (per year)	0.55	(0.0, 4.6)	0.20	(0.0, 3.4)	< 0.001
Number of visits, mean (SD) (per year)	4.76	(9.89)	4.07	(9.67)	
Pulpitis (ICD-9-CM 522.0)					
Prevalence (%)	421	(31.5)	3755	(28.1)	0.009
Number of visits, median (Q1, Q3) (per year)	0.00	(0.0, 0.8)	0.00	(0.0, 0.4)	0.002
Number of visits, mean (SD) (per year)	1.45	(4.15)	1.17	(3.61)	
Gingivitis (ICD-9-CM 523.0, 523.1, 523.2)					
Prevalence (%)	449	(33.6)	4005	(30.0)	0.006
Number of visits, median (Q1, Q3) (per year)	0.00	(0.0, 0.9)	0.00	(0.0, 0.5)	0.001
Number of visits, mean (SD) (per year)	1.45	(4.32)	1.11	(3.64)	
Periodontitis (ICD-9-CM 523.3, 523.4, 523.5, 523.8)					
Prevalence (%)	762	(57.0)	7172	(53.6)	0.019
Number of visits, median (Q1, Q3) (per year)	0.59	(0.0, 3.7)	0.28	(0.0, 3.1)	0.001
Number of visits, mean (SD) (per year)	4.46	(10.53)	3.85	(9.91)	
Oral ulceration (ICD-9-CM 528.2)					
Prevalence (%)	229	(17.1)	1775	(13.3)	< 0.001
Number of visits, median (Q1, Q3) (per year)	0.00	(0.0, 0.0)	0.00	(0.0, 0.0)	< 0.001
Number of visits, mean (SD) (per year)	0.80	(4.40)	0.52	(2.83)	
Stomatitis (ICD-9-CM 528.0)					
Prevalence (%)	124	(9.3)	1108	(8.3)	0.214
Number of visits, median (Q1, Q3) (per year)	0.00	(0.0, 0.0)	0.00	(0.0, 0.0)	0.197
Number of visits, mean (SD) (per year)	0.39	(2.01)	0.31	(2.41)	
Temporomandibular joint disorders (ICD-9-CM 524.6)					
Prevalence (%)	21	(1.6)	137	(1.0)	0.065
Number of visits, median (Q1, Q3) (per year)	0.00	(0.0, 0.0)	0.00	(0.0, 0.0)	0.065
Number of visits, mean (SD) (per year)	0.04	(0.46)	0.03	(0.46)	

*P* values were obtained by Chi-square test for comparison of prevalence and Mann–Whitney *U*-test for comparison of medians of number of visits

ICD-9-CM, International Classification of Diseases, Ninth revision, clinical modification; Q1, lower quartile; Q3, upper quartile; SD, standard deviation

### Incidence of visits for dental caries and pulpitis in patients with RA

In Table 3, we found that patients with RA had an increased incidence of visits for dental caries (adjusted IRR = 1.16, 95% CI 1.07–1.26, *p* < 0.001) compared with patients without RA. When stratified by sex, female RA patients had an elevated incidence for dental caries (adjusted IRR = 1.21, 95% CI 1.11–1.33, *p* < 0.001), but not in male RA patients (adjusted IRR = 0.98, 95% CI 0.81–1.18, *p* = 0.804). Moreover, compared with patients without RA, those with RA, both the 20–55 years age group (adjusted IRR = 1.18, CI 1.06–1.31; *p* = 0.002) and > 55 years age group (adjusted IRR = 1.18, CI 1.03–1.34, *p* = 0.014) showed an increased incidence of dental caries.

**Table 3:** Incidence and incidence rate ratios of visits for dental caries and pulpitis in the rheumatoid arthritis cohort and the comparison cohort

Disorder	Group	Rheumatoid arthritis cohort			Comparison cohort			IRR (95% CI)	aIRR (95% CI)
(ICD-9-CM)	(Years)	No. of patient	Person-years	Incidence	No. of patient	Person-years	Incidence	*p* value	*p* value
Dental caries^a^ (521.0)									
	Overall	683	4009	170.37	6085	41,693	145.95	1.17 (1.08–1.26) < 0.001	1.16 (1.07–1.26) < 0.001
	Sex								
	Male	125	1019	122.67	1275	9768	130.53	0.94 (0.78–1.13) 0.508	0.98 (0.81–1.18) 0.804
	Female	558	2990	186.62	4810	31,925	150.67	1.24 (1.14–1.35) < 0.001	1.21 (1.11–1.33) < 0.001
	Age group (years)								
	20–54	414	2112	196.02	3736	22,448	166.43	1.18 (1.06–1.30) 0.002	1.18 (1.06–1.31) 0.002
	> 55	269	1897	141.80	2349	19,245	122.06	1.16 (1.02–1.32) 0.020	1.18 (1.03–1.34) 0.014
Pulpitis^b^ (522.0)									
	Overall	405	5885	68.82	3608	58,912	61.24	1.12 (1.01–1.24) 0.026	1.12 (1.00–1.24) 0.044
	Sex								
	Male	65	1386	46.90	738	13,553	54.45	0.86 (0.67–1.11) 0.249	0.89 (0.68–1.15) 0.368
	Female	340	4499	75.57	2870	45,359	63.27	1.19 (1.07–1.34) 0.002	1.18 (1.05–1.32) 0.006
	Age group (years)								
	20–54	242	3291	73.53	2207	33,505	65.87	1.12 (0.98–1.28) 0.104	1.11 (0.96–1.27) 0.157
	> 55	163	2594	62.84	1401	25,407	55.14	1.14 (0.97–1.34) 0.115	1.15 (0.97–1.36) 0.105

Adjusted for age, sex, socioeconomic status, geographic region, hypertension, diabetes, congestive heart failure, chronic pulmonary disease, cancer, dyslipidemia, coronary artery disease, prior myocardial infarction, peripheral vascular disease, cerebrovascular disease, dementia, musculoskeletal disorders, peptic ulcer disease, chronic kidney disease, and liver disease

aIRR, adjusted incidence rate ratio; ICD-9-CM, International Classification of Diseases, Ninth revision, clinical modification; IRR, incidence rate ratio

^a^Patients who were diagnosed with dental caries within 90 days before the index date were excluded and 1248 patients with rheumatoid arthritis and 12,332 controls were included in the analysis

^b^Patients who were diagnosed with pulpitis within 90 days before the index date were excluded and 1307 patients with rheumatoid arthritis and 13,061 controls were included in the analysis

Patients with RA also showed an elevated incidence of visits for pulpitis (adjusted IRR = 1.12, 95% CI 1.00–1.24, *p* = 0.044) compared with those without RA. Female patients with RA had an elevated incidence for pulpitis (adjusted IRR = 1.18, 95% CI 1.05–1.32, *p* = 0.006), but not in male patients (adjusted IRR = 0.89, 95% CI 0.68–1.15, *p* = 0.368). The incidence for pulpitis in patients with RA was not significantly higher in different age groups.

### Incidence of visits for gingivitis and periodontitis in patients with RA

In Table 4, we found that patients with RA had a significantly higher incidence of visits for gingivitis compared to those without RA (adjusted IRR = 1.13, 95% CI 1.01–1.25, *p* = 0.027). Female patients with RA showed an increased visits for gingivitis (adjusted IRR = 1.19, 95% CI 1.06–1.34, *p* = 0.003), but male patients did not (adjusted IRR = 0.86, 95% CI 0.66–1.11, *p* = 0.241). When stratified by age group, RA patients in the younger age group (20–55 years) had an increased incidence of gingivitis (adjusted IRR = 1.17, 95% CI 1.02–1.33, *p* = 0.022), but not in the older age group (aged > 55 years; adjusted IRR = 1.09, 95% CI 0.92–1.33, *p* = 0.330).

**Table 4:** Incidence and incidence rate ratios of visits for gingivitis and periodontitis in the rheumatoid arthritis cohort and the comparison cohort

Disorder	Age group	Rheumatoid arthritis cohort			Comparison cohort			IRR (95% CI)	aIRR (95% CI)
(ICD-9-CM)	(Years)	No. of patient	Person-years	Incidence	No. of patient	Person-years	Incidence	*p* value	*p* value
Gingivitis^a^ (523.0, 523.1, 523.2)									
	Overall	419	5938	70.56	3805	60,310	63.09	1.12 (1.01–1.24) 0.030	1.13 (1.01–1.25) 0.027
	Sex								
	Male	67	1357	49.37	817	13,499	60.52	0.82 (0.64–1.05) 0.109	0.86 (0.66–1.11) 0.241
	Female	352	4581	76.84	2988	46,811	63.83	1.20 (1.08–1.34) 0.001	1.19 (1.06–1.34) 0.003
	Age group (years)								
	20–54	269	3258	82.57	2443	33,870	72.13	1.14 (1.01–1.30) 0.036	1.17 (1.02–1.33) 0.022
	> 55	150	2680	55.97	1362	26,440	51.51	1.09 (0.92–1.29) 0.334	1.09 (0.92–1.30) 0.330
Periodontitis^b^ (523.3, 523.4, 523.5, 523.8)									
	Overall	694	4049	171.40	6239	40,686	153.35	1.12 (1.03–1.21) 0.005	1.13 (1.04–1.22) 0.004
	Sex								
	Male	144	989	145.60	1381	8943	154.42	0.94 (0.79–1.12) 0.503	1.00 (0.83–1.19) 0.962
	Female	550	3060	179.74	4858	31,743	153.04	1.17 (1.08–1.28) < 0.001	1.17 (1.07–1.28) 0.001
	Age group (years)								
	20–54	405	2207	183.51	3722	22,673	164.16	1.12 (1.01–1.24) 0.033	1.15 (1.03–1.28) 0.013
	> 55	289	1842	156.89	2517	18,013	139.73	1.12 (0.99–1.27) 0.063	1.14 (1.00–1.29) 0.043

Adjusted for age, sex, socioeconomic status, geographic region, hypertension, diabetes, congestive heart failure, chronic pulmonary disease, cancer, dyslipidemia, coronary artery disease, prior myocardial infarction, peripheral vascular disease, cerebrovascular disease, dementia, musculoskeletal disorders, peptic ulcer disease, chronic kidney disease, and liver disease

aIRR, adjusted incidence rate ratio; ICD-9-CM, International Classification of Diseases, Ninth revision, clinical modification; IRR, incidence rate ratio

^a^Patients who were diagnosed with gingivitis within 90 days before the index date were excluded and 1288 patients with rheumatoid arthritis and 13,019 controls were included in the analysis

^b^Patients who were diagnosed with periodontitis within 90 days before the index date were excluded and 1248 patients with rheumatoid arthritis and 12,152 controls were included in the analysis

Patients with RA had an elevated incidence of visit for periodontitis compared with patients without RA (adjusted IRR = 1.13, 95% CI 1.04–1.22, *p* = 0.004). Moreover, female patients with RA showed a significantly increased incidence of periodontitis (adjusted IRR = 1.17, 95% CI 1.07–1.28, *p* = 0.001), but not in male patients with RA (adjusted IRR = 1.00, 95% CI 0.83–1.19, *p* = 0.962). Moreover, patients with RA showed an increased incidence of periodontitis in the 20–54 years group (adjusted IRR = 1.15, 95% CI 1.03–1.28, *p* = 0.013) and the > 55 years group (adjusted IRR = 1.14, 95% CI 1.00–1.29, *p* = 0.043).

### Incidence of visits for oral ulceration and temporomandibular joint disorder in patients with RA

In Table 5, we found that patients with RA had an elevated incidence of oral ulceration compared with patients without RA (adjusted IRR = 1.24, 95% CI 1.08–1.44, *p* = 0.003). Female RA patients with RA showed an elevated incidence for having oral ulceration (adjusted IRR = 1.29, 95% CI 1.10–1.51, *p* = 0.002), but not in male patients with RA (adjusted IRR = 1.05, 95% CI 0.73–1.50, *p* = 0.804). When stratified by age, the younger group patients with RA showed an elevated incidence of oral ulceration (adjusted IRR = 1.30, 95% CI 1.06–1.58, *p* = 0.011), but not those with older age group (adjusted IRR = 1.21, CI 0.98–1.50, *p* = 0.083).

**Table 5:** Incidence and incidence rate ratios of visits for oral ulceration and temporomandibular joint disorders in the rheumatoid arthritis cohort and the comparison cohort

Disorder	Age group (years)	Rheumatoid arthritis cohort			Comparison cohort			IRR (95% CI)	aIRR (95% CI)
(ICD-9-CM)		No. of patient	Person-years	Incidence	No. of patient	Person-years	Incidence	*p* value	*P* value
Oral ulceration^a^ (528.2)									
	Overall	222	7057	31.46	1734	70,434	24.62	1.28 (1.11–1.47) 0.001	1.24 (1.08–1.44)0.003
	Sex								
	Male	34	1539	22.09	351	15,880	22.10	1.00 (0.70–1.42) 0.999	1.05 (0.73–1.50) 0.804
	Female	188	5518	34.07	1383	54,554	25.35	1.34 (1.15–1.56) < 0.001	1.29 (1.10–1.51) 0.002
	Age group (years)								
	20–54	121	4117	29.39	934	41,241	22.65	1.30 (1.07–1.57) 0.007	1.30 (1.06–1.58) 0.011
	> 55	101	2940	34.35	800	29,193	27.40	1.25 (1.02–1.54) 0.032	1.21 (0.98–1.50) 0.083
Temporomandibular joint disorders^b^ (524.6)									
	Overall	18	7697	2.34	117	76,580	1.53	1.53 (0.93–2.51) 0.093	1.31 (0.77–2.23) 0.320
	Sex								
	Male	2	1660	1.20	23	17,083	1.35	0.90 (0.21–3.80) 0.881	0.79 (0.18–3.42) 0.750
	Female	16	6037	2.65	94	59,497	1.58	1.68 (0.99–2.85) 0.056	1.33 (0.75–2.36) 0.327
	Age group (years)								
	20–54	11	4448	2.47	70	44,502	1.57	1.57 (0.83–2.97) 0.163	1.40 (0.72–2.76) 0.323
	> 55	7	3249	2.15	47	32,078	1.47	1.47 (0.66–3.25) 0.341	1.24 (0.52–2.96) 0.630

Adjusted for age, sex, socioeconomic status, geographic region, hypertension, diabetes, congestive heart failure, chronic pulmonary disease, cancer, dyslipidemia, coronary artery disease, prior myocardial infarction, peripheral vascular disease, cerebrovascular disease, dementia, musculoskeletal disorders, peptic ulcer disease, chronic kidney disease, and liver disease

aIRR, adjusted incidence rate ratio; ICD-9-CM, International Classification of Diseases, Ninth revision, clinical modification; IRR, incidence rate ratio

^a^Patients who were diagnosed with oral ulceration within 90 days before the index date were excluded and 1326 patients with rheumatoid arthritis and 13,275 controls were included in the analysis

^b^Patients who were diagnosed with temporomandibular joint disorders within 90 days before the index date were excluded and 1302 patients with rheumatoid arthritis and 13,229 controls were included in the analysis

Furthermore, no significant differences were observed in developing temporomandibular joint disorders between patients with and without RA either in the overall analysis (adjusted IRR = 1.31, 95% CI 0.77–2.23, *p* = 0.320) or in the subgroup analyses stratified by sex or age group.

## Discussion

In this secondary cohort study, we found the prevalence and frequency of dental visits were increased in patients with RA. The incidence of visits for dental caries, pulpitis, gingivitis, periodontitis and oral ulcer, but not temporomandibular joint disorders was significantly higher in patients with RA especially female patients. For age group, the risks of dental caries and periodontitis were increased in both the younger and older age groups and the risk of gingivitis and oral ulcer were increased in younger age group.

Dental caries is a biofilm-mediated disease with multiple contributing factors that drives net localized demineralization of the teeth [14]. Although RA is considered a risk factor for dental caries, few studies have shown that the frequency and severity of dental caries were also higher in patients with RA [9, 15]. It is interesting to note that RA disease activity could be associated with the severity of dental caries [15]. It should be noted that primary Sjögren's syndrome is a strong risk factor for developing dental caries (aIRR = 1.64) according to our previous study [12]. Therefore, patients with a diagnosis of primary Sjögren's syndrome were excluded from this study. However, patients with RA might still have secondary Sjögren's syndrome. Therefore, it is possible that the developing of dental caries and pulpitis were mediated through hyposalivation in patients with RA [16].

There is a very close and complex relationship between periodontitis and RA. Both periodontitis and RA shared similar pathogenesis, including imbalance between pro- and anti-inflammatory cytokines, the role of smoking, microbial dysbiosis especially *Porphyromonas gingivalis,* and genetic background [17]. Therefore, our finding was as expected. Periodontitis, a major inflammatory disease of the oral mucosa, is epidemiologically associated with other chronic inflammation-driven disorders, including cardiometabolic, neurodegenerative and autoimmune diseases, and cancer [18]. A recent systematic review analysis of eight articles on nonsurgical treatment of patients with periodontitis found that periodontitis treatment could improve clinical and laboratory test parameters, such as disease activity score by 28 joints [DAS28] and erythrocyte sedimentation rate, of RA [19]. The causal relationship of periodontitis and RA remained to be elucidated. The incidence for oral ulceration was increased in patients with RA, and it could be related to the side effect of common medication methotrexate in the treatment of RA [20]. It is known that RA could destruct the temporomandibular joint [21]. Byun et al. reported a 2.52-fold increased risk of developing temporomandibular joint disorder in patients with RA in a cohort study of 3122 patients with RA and 12,488 matched controls [22]. The sample size of patients with RA was 1337 in the present study, and this smaller sample size might explain why only a trend of increased risk of developing temporomandibular joint disorder was observed in patients with RA.

Overall the findings of this secondary cohort study indicated that patients with had a poor oral health condition, which is similar to a Korean study [23]. Poor oral health might increase personal medical expenses and burden of health insurance. In contrast, good oral health might be a starting point for the general health and well-being of our body [24]. Rheumatologists should be vigilant for the dental condition in their patients with RA [25] and dental professionals should be aware for the potential poor oral health in patients with RA and effect should be made to enhance their oral health.

The strengths of this study is the large number of patients with RA (n = 1337) and a 12-year follow up period. All the diagnoses were made by specialists and the study sample was representative for the whole population in Taiwan. Nevertheless, there are some limitations. First, identification of dental disorders was based on the claim data, some patients with only mild dental disorders might not visit dental professionals. Second, due to the limitation of claim data, we did not have information on the severity of RA and dental disorders. Third, the database used in this study spanned from January 2000 to December 2012, whether the current situation remained the same will require confirmation.

In conclusion, we found an elevated prevalence and frequency of dental visits in patients with RA. In addition, elevated incidence of visits for dental disorders, including dental caries, pulpitis, gingivitis, periodontitis, and oral ulceration, were observed. Both the rheumatologist and dental professionals should accessed the oral health condition regularly in patients with RA.

## Data Availability

The data that support the findings of this study are available on request from the corresponding author. The data are not publicly available due to the Taiwan Personal Information Protection Act.

## References

[1] Kapila YL (2000). Oral health's inextricable connection to systemic health: Special populations bring to bear multimodal relationships and factors connecting periodontal disease to systemic diseases and conditions. Periodontol.

[2] Scott DL, Wolfe F, Huizinga TW (2010). Rheumatoid arthritis. Lancet.

[3] Chou CT, Pei L, Chang DM, Lee CF, Schumacher HR, Liang MH (1994). Prevalence of rheumatic diseases in Taiwan: a population study of urban, suburban, rural differences. J Rheumatol.

[4] Smolen JS, Aletaha D, McInnes IB (2016). Rheumatoid arthritis. Lancet.

[5] Figus FA, Piga M, Azzolin I, McConnell R, Iagnocco A (2021). Rheumatoid arthritis: extra-articular manifestations and comorbidities. Autoimmun Rev.

[6] de Souza S, Bansal RK, Galloway J (2016). Rheumatoid arthritis - an update for general dental practitioners. Br Dent J.

[7] Chen HH, Huang N, Chen YM, Chen TJ, Chou P, Lee YL (2013). Association between a history of periodontitis and the risk of rheumatoid arthritis: a nationwide, population-based, case-control study. Ann Rheum Dis.

[8] Tang Q, Fu H, Qin B, Hu Z, Liu Y, Liang Y (2017). A possible link between rheumatoid arthritis and periodontitis: a systematic review and meta-analysis. Int J Periodontics Restorative Dent.

[9] Martinez-Martinez RE, Domínguez-Pérez RA, Sancho-Mata J, Abud-Mendoza C, Ayala-Herrera JL, Popoca-Hernandez EA (2019). The frequency and severity of dental caries, and counts of cariogenic bacteria in rheumatoid arthritis patients. Dent Med Probl.

[10] Lin CY, Chung CH, Chu HY, Chen LC, Tu KH, Tsao CH (2017). Prevalence of temporomandibular disorders in rheumatoid arthritis and associated risk factors: a nationwide study in Taiwan. J Oral Facial Pain Headache.

[11] Kroese JM, Volgenant CMC, Crielaard W, Loos B, van Schaardenburg D, Visscher CM (2021). Temporomandibular disorders in patients with early rheumatoid arthritis and at-risk individuals in the Dutch population: a cross-sectional study. RMD Open.

[12] Chuang CJ, Hsu CW, Lu MC, Koo M (2020). Increased risk of developing dental diseases in patients with primary Sjögren's syndrome-A secondary cohort analysis of population-based claims data. PLoS ONE.

[13] Arnett FC, Edworthy SM, Bloch DA, McShane DJ, Fries JF, Cooper NS (1988). The American Rheumatism Association 1987 revised criteria for the classification of rheumatoid arthritis. Arthritis Rheum.

[14] Sabharwal A, Stellrecht E, Scannapieco FA (2021). Associations between dental caries and systemic diseases: a scoping review. BMC Oral Health.

[15] Äyräväinen L, Heikkinen AM, Kuuliala A, Ahola K, Koivuniemi R, Peltola J (2018). Activity of rheumatoid arthritis correlates with oral inflammatory burden. Rheumatol Int.

[16] Pedersen AML, Sørensen CE, Proctor GB, Carpenter GH, Ekström J (2018). Salivary secretion in health and disease. J Oral Rehabil.

[17] de Molon RS, Rossa C, Thurlings RM, Cirelli JA, Koenders MI (2019). Linkage of periodontitis and rheumatoid arthritis: current evidence and potential biological interactions. Int J Mol Sci.

[18] Hajishengallis G, Chavakis T (2021). Local and systemic mechanisms linking periodontal disease and inflammatory comorbidities. Nat Rev Immunol.

[19] Silvestre FJ, Silvestre-Rangil J, Bagán L, Bagán JV (2016). Effect of nonsurgical periodontal treatment in patients with periodontitis and rheumatoid arthritis: a systematic review. Med Oral Patol Oral Cir Bucal.

[20] Nakarmi S, Pudasaini K, Adhikari B, Vaidya B (2020). Adverse events profile of low-dose methotrexate in nepalese patients with rheumatoid arthritis: an observational study. J Nepal Health Res Counc.

[21] Campos DES, de Araújo Ferreira-Muniz I, de Souza Villarim NL, Ribeiro ILA, Batista AUD, Bonan PRF (2021). Is there an association between rheumatoid arthritis and bone changes in the temporomandibular joint diagnosed by cone-beam computed tomography? A systematic review and meta-analysis. Clin Oral Investig.

[22] Byun SH, Min C, Choi HG, Hong SJ (2020). Increased risk of temporomandibular joint disorder in patients with rheumatoid arthritis: A longitudinal follow-up study. J Clin Med.

[23] Shim H, Koo J, Ahn J (2022). Association between rheumatoid arthritis and poor self-perceived oral health in Korean adults. Healthcare (Basel).

[24] Fiorillo L (2019). Oral health: the first step to well-being. Medicina (Kaunas).

[25] Treister N, Glick M (1999). Rheumatoid arthritis: a review and suggested dental care considerations. J Am Dent Assoc.

